# Dry Sliding Wear and Corrosion Performance of Mg-Sn-Ti Alloys Produced by Casting and Extrusion

**DOI:** 10.3390/ma15103533

**Published:** 2022-05-14

**Authors:** Davoud Bovand, Hassan Abdollah-Pour, Omid Mirzaee, Sara Bagherifard

**Affiliations:** 1 Faculty of Materials and Metallurgical Engineering, Semnan University, Semnan 35131-19111, Iran; davood_bovand@yahoo.com (D.B.); o_mirzaee@semnan.ac.ir (O.M.); 2 Department of Mechanical Engineering, Politecnico di Milano, 20156 Milan, Italy

**Keywords:** magnesium–Ti alloys, corrosion, wear properties, extrusion

## Abstract

The aim of the present study is to investigate the role of Ti on corrosion and the wear properties of Mg-5Sn-xTi (x = 0, 0.15, 0.75, 1.5 wt.%) alloys. The samples were fabricated by conventional casting followed by hot extrusion, and the studies were examined by means of a pin-on-disc tribometer at various loads of 6, 10, and 20 N with constant sliding velocities of 0.04 m/s at ambient temperature. The corrosion performance, using potentiodynamic polarization and electrochemical impedance spectroscopy (EIS), was studied in a basic solution containing 3.5 wt.% NaCl. The observation indicated a drop in the wear rate with an increase in Ti, while the average coefficient of friction was raised in higher Ti contents compared to the base material. The sample with 0.75 wt.% Ti exhibited superior wear properties at 6 and 10 N of normal force, while the sample with 0.15 wt.% Ti presented better wear resistance for 20 N. Electrochemical test observations demonstrated that the Ti deteriorated the corrosion features of the Mg-5Sn alloy, owing to the galvanic effects of Ti. The Mg-5Sn alloy exhibited excellent corrosion behavior (corrosion potential (E_corr_) = −1.45V and current density (I_corr_) = 43.92 A/cm^2^). The results indicated the significant role of Ti content in modulating wear and corrosion resistance of the Mg-5Sn alloy.

## 1. Introduction

Magnesium alloys are receiving substantial consideration, especially because of the high request for weight reduction in different applications. Mg possesses a low weight and high specific strength among usual engineering alloys. Moreover, Mg alloys are used for their lightweight structure, industrial parts, special dimensional stability, significant damping capacity, and good recyclability [1,2,3]. However, Mg is limited in its industrial application due to its poor mechanical features, including hardness, wear, fatigue, and—above all—inadequate corrosion resistance [4,5].

The wear phenomenon, as one of the most common industrial problems, is one of the main reasons for replacing worn components with new ones over time. The energy loss caused by friction and wear could be decreased by surface modifications, developing new materials or lubrication technologies [6]. However, poor wear resistance explains the limited application of Mg and its alloys. Recently, scholars have studied the improvements in the wear characteristics of Mg alloys by composition variation, alloying, and surface modification [7,8,9,10].

Extensive studies on Mg-Sn-based alloys imply their potential as promising candidates for heat resistance applications, owing to the great solubility of Sn in α-Mg that decreases extensively from 3.35% at eutectic transformation temperature to ~ zero at room temperature to form a Mg_2_Sn phase with good thermal stability. The precipitation of the Mg_2_Sn phase with a melting point of 770 °C could efficiently serve as an obstacle to dislocation movement and could restrain grain growth to promote mechanical characteristics in both room and high temperatures. Multiple studies have reported that adding Sn diminishes corrosion rate and promotes the wear features of the alloy [11,12]. Liu et al. [13] described that the tensile strength, elongation, hardness, and creep properties of Mg-Sn alloys were strongly varied by Sn content, and that superior mechanical properties were achieved in the Mg-5 wt.% Sn alloy, showing high strength, increased elongation, as well as better hardness and creep properties.

Up to now, many studies have focused on the mechanical, microstructure, and corrosion characteristics of Mg-Sn alloys. Mechanical features of the alloys at various temperatures can be promoted through the addition of alloying elements and, subsequently, the utilization of extrusion, rolling, and forging processes [14,15,16]. Moreover, the extrusion process is an effective strategy in the grain refinement of alloys and in dispersing the Mg_2_Sn phase in related alloys, resulting in improved tensile and compressive strength. The previous investigations on Mg-Sn systems indicated that micro alloying by Zn, Y, Gd, Ca, Mn, Zr, Li, Ti, etc., improved the hardness and strength of the Mg-Sn alloy by refining the distribution, raising the volume fraction of the precipitations, and inducing grain refinement in α-Mg. Moreover, the results show that Ti could effectively refine the grains and lead to improved mechanical properties in terms of ultimate tensile strength, ultimate compressive strength, and Mg-5Sn alloy hardness [17,18,19,20,21,22]. Regarding the effect of Ti addition on the mechanical response of the material, our previous study [22] indicated that the addition of 0.15 wt.% Ti helped to improve both UTS and elongation in relation to the failure of 287 ± 3 MPa and 5.8 ± 0.4%, in comparison to the Mg-5Sn alloy with 269 ± 4 MPa and 5.6 ± 0.5%, respectively. The sample with 0.75 wt.% Ti showed the maximum UTS of 296 ± 6 MPa, whereas for 1.5 Ti the lowest UTS and elongation of 263 ± 10 MPa and 3.7 ± 0.6% were obtained, respectively. Our previous study on texture and grain orientation has shown that the addition of Ti can decrease (002) plane texture and change the preferred orientation from (002) to (101) and (100). The addition of Ti results in basal texture weakening and subsequently improves the formability of the Mg-5Sn alloy. The change in texture of the Mg-5Sn alloy could be attributed to a decrease in the generalized stacking fault energy (GSFE) on different crystallographic planes [22,23]. Micro-alloying and developing Mg-based composites can efficiently enhance mechanical characteristics; recent studies have shown that introducing the particulate reinforcements can improve the mechanical and wear properties of the Mg alloy [24,25,26,27,28]. It has been also reported that thermal stability, intermetallic phases, high-temperature performance, contact surface temperature, reinforcement size, and distribution could affect the wear resistance of Mg alloys [29].

However, very few studies have investigated the wear and corrosion behaviors of Mg-Sn-Ti alloys. Furthermore, materials, especially metals and alloys in industrial applications, are also subjected to wear and corrosion, which can simultaneously necessitate improved wear and corrosion properties. At the present study, Mg–5 wt.% Sn alloys with high Ti contents were investigated to understand the role of Ti in wear mechanisms, as well as the friction properties and the corrosion behavior of these alloys.

## 2. Materials and Methods

The Mg_95−x_Sn_5_Ti_x_ (x = 0, 0.15, 0.75, 1.5 wt.%) alloy samples were fabricated by melting commercial-purity Mg ingot (99.5%), pure Sn (99.99%), and Ti particles with an average size of 45 micron purchased from Merck KGaA (Darmstadt, Germany). First, the Mg was melted in an induction furnace with stainless steel crucible under the continuous flow of argon gas passed through the melt to prevent the alloy from oxidizing during melting. The preheated Sn and Ti were poured to the molten Mg at 790 °C and stirred at 780 °C with a rotation speed of 300 revolutions per minute using a motor-driven stirrer connected to a stainless steel blade submerged in the melt for 15 min. The melt was casted into a cylindrical stainless steel mold with a D_in_ of 40 mm, preheated to 250 °C. Due to the reduction of Mg during melting, 5 wt.% of Mg was added to the melt to ensure the desired composition. The composition of the cast samples was evaluated using an X-ray fluorescence (XRF) analyzer (S8 TIGER, Bruker, Billerica, MA, USA), as reported in Table 1.

**Figure 1:**
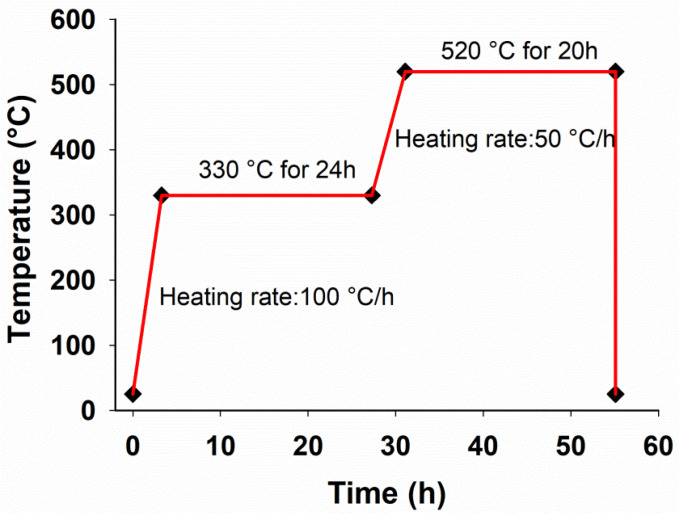
The thermal cycle of solution treatment process considered for Mg-5Sn-xTi (x = 0, 0.15, 0.75, 1.5 wt.%) alloys.

The hot extrusion process was conducted by preparing a series of samples with cylindrical shape (35 mm diameter and 23 mm height). According to the second-phase nature and the appropriate melting point, the solution treatment was applied, as shown in Figure 1, followed by quenching in water. Next, the samples were preheated at 300 °C for 15 min, and the extrusion test was then carried out with an extrusion ratio of 6:1 at a ram velocity of 60 mm/min.

The surface chemical compositions of the specimen were examined by energy-dispersive X-ray analysis (EDXA), and their morphology was illustrated by scanning electron microscopy (SEM) (LEO-1455VP, Firma Zeiss, Oberkochen, Germany) equipped with an energy-dispersive X-ray spectroscopy. Before SEM analysis, the samples were placed on aluminum stubs using a conductive strip, and then coated with gold. A micro-Vickers hardness machine (Buehler Co., Braunschweig, Germany), with a load of 200 gf and a dwell time of 15 s, was utilized for measuring the Vickers microhardness of the polished bulk specimens. To ensure data reliability, the hardness tests were repeated 4 times for each sample. The phase constituent of extruded samples was determined by X-ray diffraction (XRD) (XRD, BRUKER D8, Karlsruhe, Germany, Cu Kα radiation, λ = 0.154056 nm).

The wear samples were prepared by machining the extruded Mg-5Sn-XTi alloy to pellets of 12 and 5 mm diameter and thickness, respectively. The samples were polished using 2000 SiC grit paper, rinsed with acetone, and dried at 50 °C. Dry sliding wear analysis was conducted by means of a pin-on-disk wear test machine using a carbon chromium steel pin with a hardness of 60 HRC under different normal loads of 6, 10, and 20 N at a sliding velocity of 0.04 m/s, and all tests conducted at room temperature. The weight-loss test was applied to examine the wear rate of alloys by measuring the specimen’s weight before and after the experiment on an electronic weighing pan with a precision of 0.001 g.

Specimens for electrochemical tests were prepared by machining the extruded samples in pellet form with 14 mm diameter and 5 mm thickness. Prior to tests, the samples were polished with 2000 SiC grit and rinsed with acetone to avoid any contamination. The change in the open circuit potential (OCP) as a function of immersion time was first measured for about 30 min. Potentiodynamic polarization analysis was carried out from −0.3 to 6.0 v against the OCP, using a scan speed of 1 mv/s for all specimens. To investigate the electrochemical performance of the Mg-5Sn-xTi alloys, the electrochemical impedance spectroscopy (EIS) evaluations were investigated by a conventional three-electrode cell setup with a platinum wire as the counter electrode, Ag/AgCl as a reference, and the Mg-5Sn-xTi alloys as the working electrode. A sinusoidal perturbation amplitude of 10 mv from 10^−2^ to 10^5^ Hz was considered. The process was performed at ambient temperature (25 °C) for all samples. To investigate the corrosion performance of the samples, an immersion test was performed using a 3.5 wt.% NaCl solution with an immersion time of 10 min and 2 h at room temperature, and the microstructure of the corroded surfaces was investigated by SEM and optical microscopy (Olympus model).

## 3. Results and Discussion

### 3.1. Microstructure and XRD Analysis

**Figure 2:**
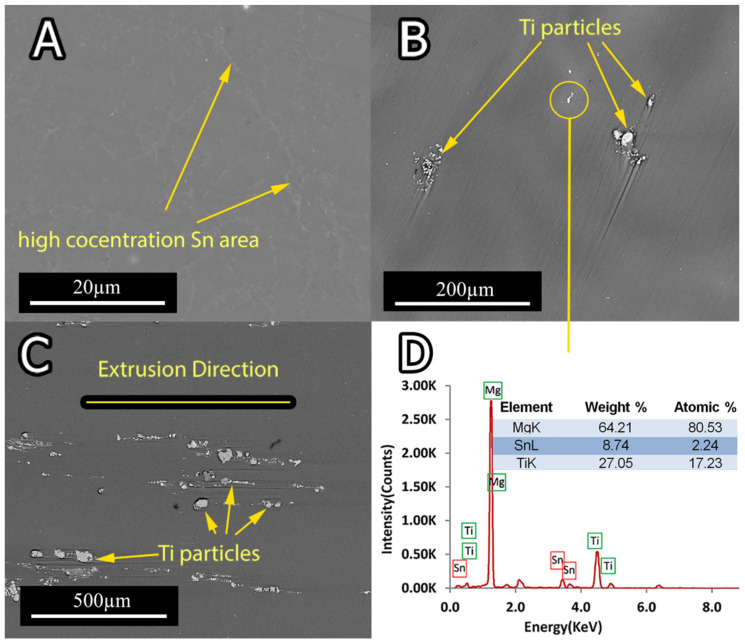
SEM micrograph of (**A**) the 0.15Ti sample perpendicular to the extrusion direction; (**B**) the 0.75Ti sample perpendicular to the extrusion direction; (**C**) the 1.5Ti sample along the extrusion direction; and (**D**) the EDXA spectrum of the selected area in (**B**).

Prior to extrusion, all the samples were subjected to two-stage solution treatment. Based on the Mg-Sn binary-phase diagram, the solution treatment peak temperature was set to 520 °C [30]. From Figure 2A, it can be seen that the peak temperature of 520 °C and the holding time of 20 h were high enough to dissolve all the Sn in the Mg matrix and form the single supersaturated Mg phase. Figure 2 demonstrates the SEM micrographs of the solutionized and extruded samples. In Figure 2A–C, for all the samples, the Mg_2_Sn phase mainly went into solid solution. All samples exhibited two distinct gray and white areas, which represent Mg-rich and Sn-rich areas, respectively. The Sn-rich area was formed during the hot extrusion process as a result of the segregation of Sn in the interdendritic area. Figure 2C exhibits Ti particles broken into fragments and aligned along the extrusion direction. During the solution treatment, the Ti particles transformed into SnTi_3_ phases according to the EDS analysis (Figure 2D).

**Figure 3:**
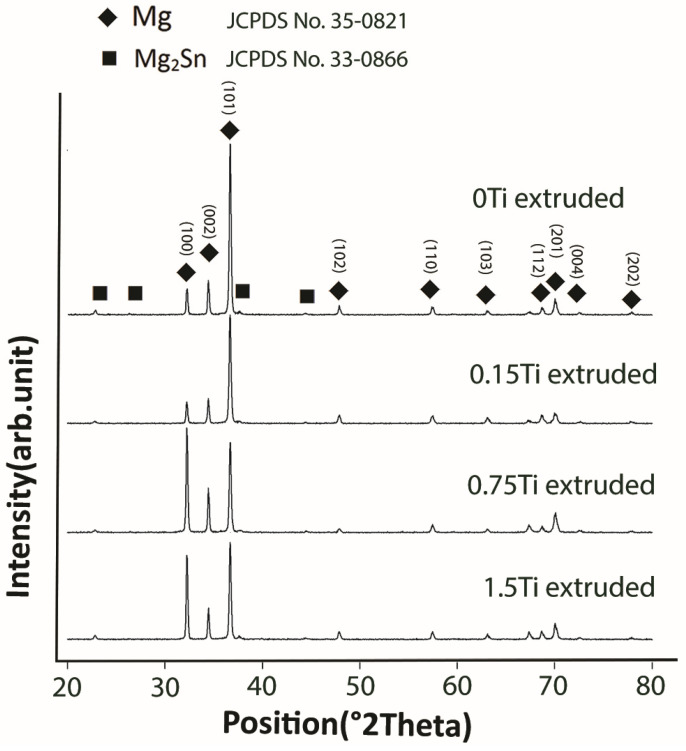
The XRD pattern of extruded Mg-5Sn-xTi samples.

Figure 3 shows the XRD pattern of extruded Mg-5Sn-xTi (x = 0, 0.15, 0.75, 1.5 wt.%) alloys. The XRD pattern shows only α-Mg as the major phase, together with a weak peak, corresponding to the Mg_2_Sn phase; no peaks related to the Ti or Ti compound were found, which can be due to the low volume fraction of Ti that could not be detected by XRD measurements. The weak peak corresponding to the Mg_2_Sn phase could be attributed to the precipitation of Mg_2_Sn during hot extrusion at 300 °C. Moreover, the 1.5Ti sample shows a more intense diffraction peak of the Mg_2_Sn phase, indicating the effect of Ti on facilitating Mg_2_Sn precipitation during hot extrusion.

### 3.2. Wear Behavior

**Figure 4:**
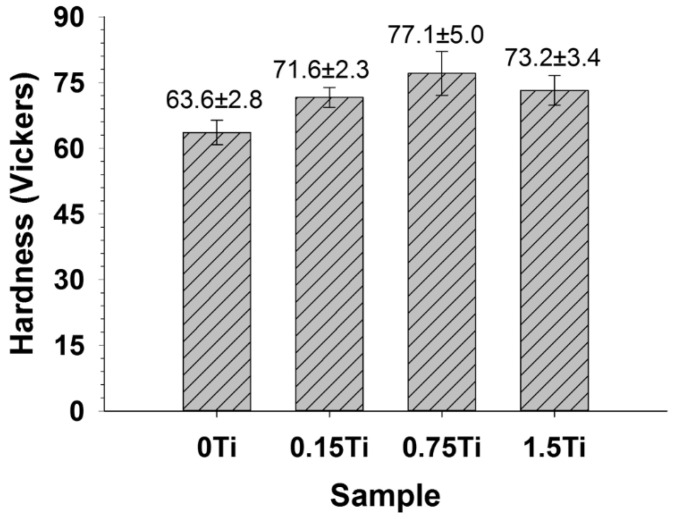
The average Vickers microhardness (mean ± STD value) measured on the samples.

Figure 4 illustrates the average microhardness of the extruded Mg-5Sn-xTi samples. The obtained results indicated that Ti enhanced the hardness of the Mg-5Sn alloys. The results show that the addition of Ti led to an increase in hardness of the Mg-5Sn alloy from 63.6 ± 2.8 HV to 71.6 ± 2.3 HV for samples with 0 and 0.15 wt.% Ti, respectively. The sample with 0.75 wt.% shows the highest hardness of 77.1 ± 5.0; the hardness of the sample with 1.5 wt.% Ti slightly decreases to 73.2 ± 3.4 HV, which can be related to the non-uniform distribution of Ti/SnTi_3_ particles in the Mg-5Sn matrix. However, the variation between the microhardness in different samples is not significant. It must be noted that increase in the microhardness of Mg-5Sn-xTi can be attributed to (i) solution hardening induced by Ti atoms and (ii) the strengthening effect due to the presence of Ti/SnTi_3_ particles, which were distributed in the Mg-5Sn matrix. Owing to the low solubility of Ti in the Mg alloy, it cannot have any significant impact on the strengthening of the Mg-5Sn alloy through the solid solution mechanism; thus, it can be concluded that second-phase strengthening induced by Ti particles acts as a major mechanism to increase the hardness of alloys.

**Figure 5:**
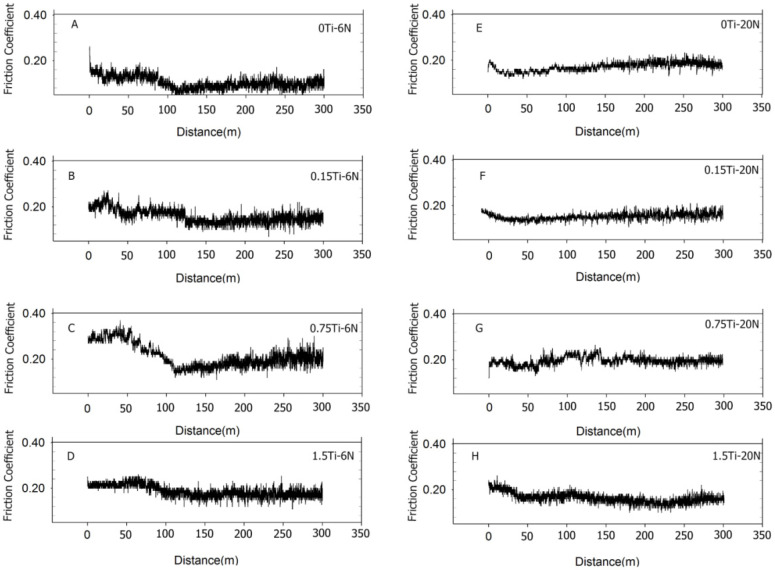
Representative graphs of difference of friction coefficient as a function of sliding distance for 6 and 20 N normal forces; (**A**) the 0Ti sample at 6 N normal force; (**B**) the 0.15Ti sample at 6 N normal force; (**C**) the 0.75Ti sample at 6 N normal force; (**D**) the 1.5Ti sample at 6 N normal force; (**E**) the 0Ti sample at 20 N normal force; (**F**) the 0.15Ti sample at 20 N normal force; (**G**) the 0.75Ti sample at 20 N normal force; and (**H**) the 1.5Ti sample at 20 N normal load.

**Figure 6:**
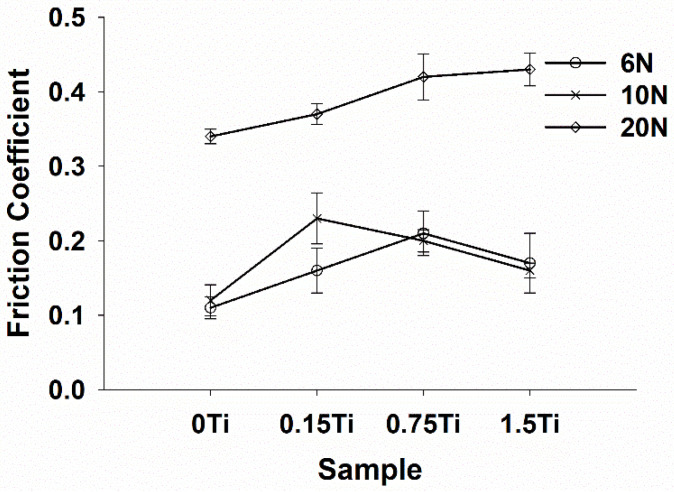
The difference of friction coefficients as a function of the normal loads for Mg-5Sn-xTi samples.

**Figure 7:**
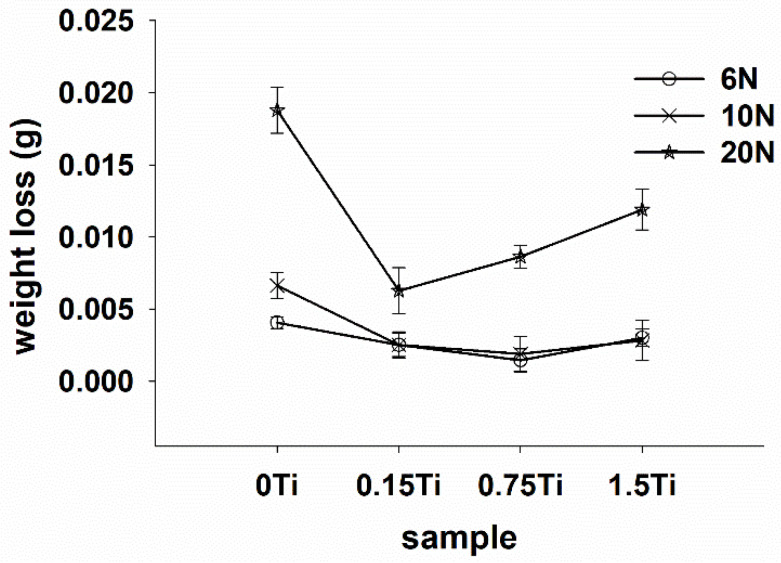
The difference of the average wear loss as a function of the normal loads for Mg-5Sn-xTi samples.

Figure 5 and Figure 6 present the representative friction coefficients of Mg-5Sn-xTi samples as a function of sliding distance and the mean friction coefficients obtained at different normal forces, respectively. The results indicate that the friction coefficient showed a higher value for all samples at 20 N normal force in comparison to the cases under 6 and 10 N normal forces. Figure 5 shows that all samples exhibited a high variation of friction coefficient at 6 N normal force due to the stick-slip behavior, which resulted in an increase in real contact; this subsequently increased the tangential force through junction growth between the pin and worn surface. In stick-slip phenomenon, the alloy surface remained in steak until the applied force exceeded the adhesive strength and ruptured the junction, leading to fast slip. Repetition of the stick-slip cycle was responsible for the variation in the friction coefficient. According to Figure 5E–H, as the forces increased, the amplitude of variation of friction coefficient decreased. From Figure 6 and Figure 7, it could be observed that all the samples containing Ti represented higher friction coefficients, while their weight loss decreased in comparison to the 0Ti sample. A review of the literature shows that introducing the particulate reinforcement leads to an enhanced wear resistance for the alloys [31,32,33]. Figure 7 indicates that the weight loss of samples decreased as the Ti content was increased. The addition of Ti from 0 to 0.75 wt.% resulted in decreased weight loss, while further addition of Ti deteriorated the wear properties of the alloy. The 1.5Ti showed a higher weight loss compared to the 0.75Ti sample at 6 and 10 N and compared to the 0.15Ti and 0.75Ti samples at 20 N normal force. An increase in the weight loss of 1.5Ti can be attributed to the detachments of Ti particles from the Mg matrix; in other words, the clustering and fracture of Ti particles (Figure 2C) due to the extrusion process could facilitate the Ti particle detachment from the Mg-5Sn matrix during wear tests; this can be more significant under higher frictional forces. In the case of 20 N normal force, due to the high shearing stress, the Ti particle broke and detached from the Mg-5Sn matrix, resulting in an increase in the weight loss of the alloy. Another aspect is the role of the protruded Ti particles, which can reenter to the matrix and finally detach from the wear surfaces due to a reduction in the area, and can consequently deteriorate the wear properties of the alloy [34]. Apart from the clustering of Ti/SnTi_3_ particles, the interfacial binding of SnTi_3_/Mg-5Sn has a notable effect on the wear properties of Mg-5Sn-xTi alloys. Good bonding can prevent the particle pull out; however, in this study, the formation of the SnTi_3_ phase on the outer layer of Ti particles (Figure 2B,C) could encourage particle detachment and thus deteriorate the wear features of the samples.

Figure 6 shows that, at all normal loads, the 0Ti represented the lowest friction coefficient. The friction coefficient of 0.75Ti and 1.5Ti at 6 and 10 N normal forces did not change significantly and were almost similar; however, at 20 N normal force, the friction coefficient of the alloys constantly increased as the Ti content was enhanced. The study conducted by Bowden and Tabor [35] showed that the coefficient of friction can be considered to have two major components: (1) the adhesion part (µ_a_) and (2) the plowing part (µ_b_).

The adhesion component is related to the material pairs and the real area of contact. On the other hand, the plowing component is affected by the extent of plastic deformation at the asperity scale. When the hard pin flows over the soft Mg-5Sn-xTi surfaces, the shear stress causes failure in the asperity level and results in an increase in the plowing component, thus increasing the coefficient of friction. Higher surface roughness leads to an increase in the plowing component and subsequently increases the coefficient of friction [36]. Due to the breakage of Ti particles and the brittle phase of SnTi_3_ during the pin slip, the surface roughness will increase and will, in turn, promote the plowing component of the friction coefficient.

**Figure 8:**
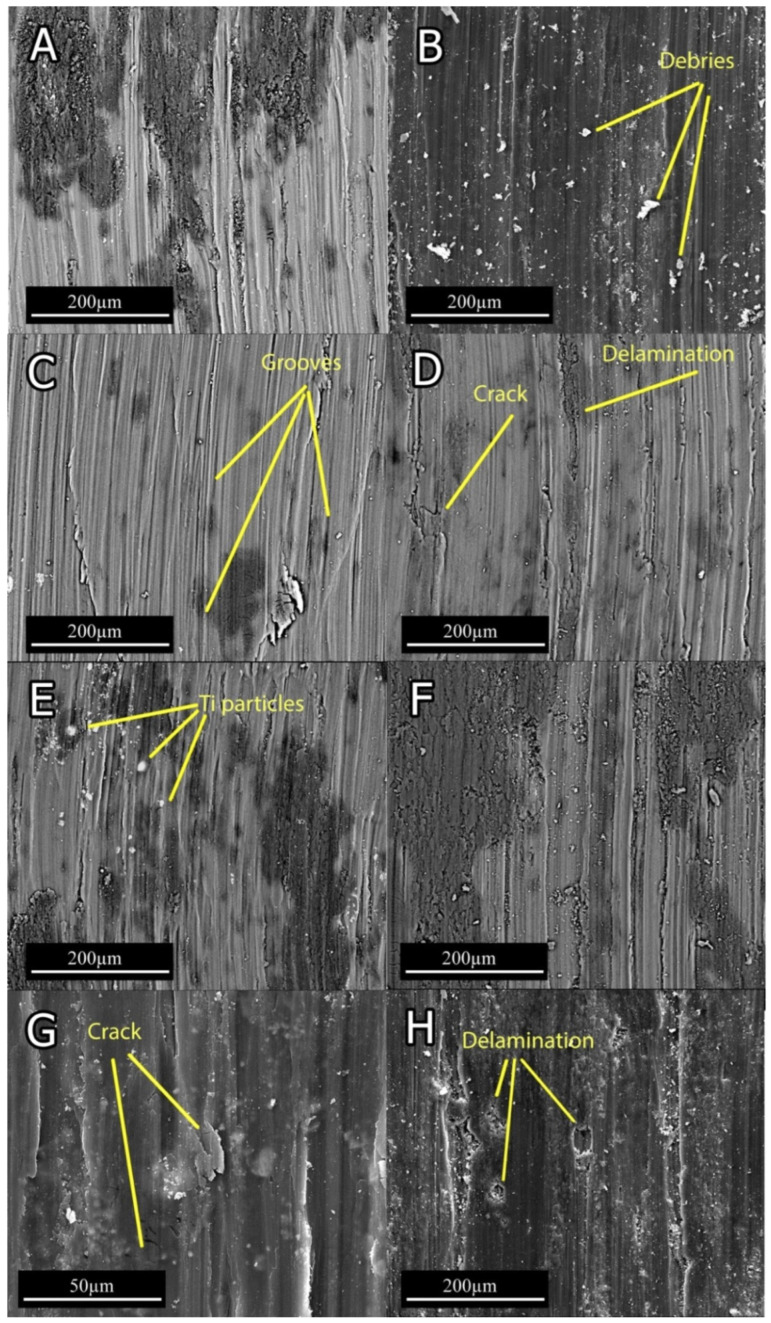
The SEM micrograph of worn surfaces at 6 and 20 N normal loads: (**A**) 0Ti at 6 N; (**B**) 0Ti at 20 N; (**C**) 0.15Ti at 6 N; (**D**) 0.15Ti at 20 N; (**E**) 0.75Ti at 6 N; (**F**) 0.75Ti at 20 N; (**G**) 1.5Ti at 6 N; and (**H**) 1.5Ti at 20 N.

Figure 8 demonstrates the SEM micrograph of worn surfaces. All specimens show a combination of grooves, which indicate abrasion mechanism and the ripped plate associated with the delamination mechanism. The grooves that are elongated in wear direction (Figure 8A,C) indicate that the dominant wear mechanism at 6 N normal force for 0Ti and 0.15Ti was abrasion. In 0Ti and 0.15Ti at 20 N normal load (Figure 8B,D), small peeled-out regions were detected. This observation implies that the contribution of delamination mechanism increased under higher normal loads. In light of observing 0Ti and 0.15Ti samples, the smooth continuous grooves became deeper and the damaged region was extended at higher applied normal loads.

**Figure 9:**
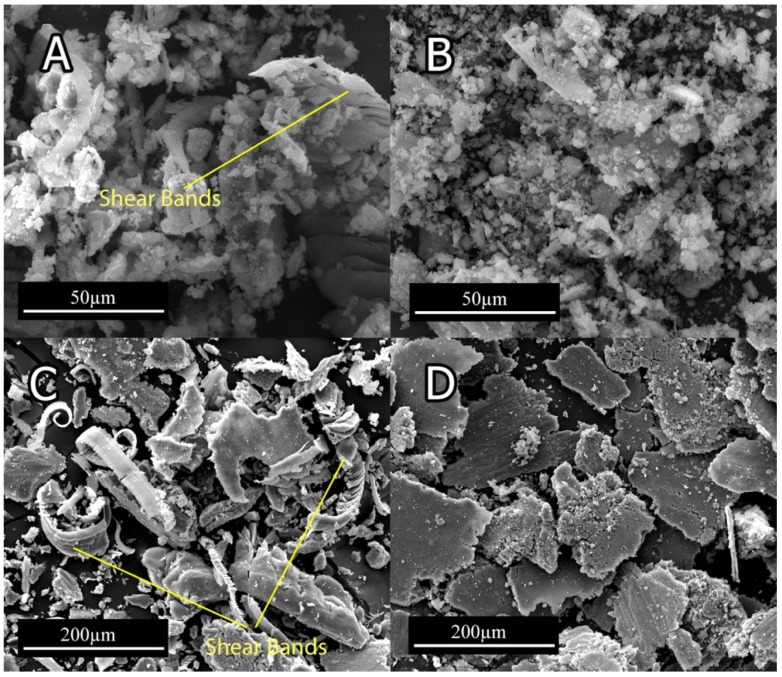
The wear debris of (**A**) 0Ti at 6 N; (**B**) 0.15Ti at 6 N; (**C**) 0.75Ti at 20 N; and (**D**) 1.5Ti at 20 N.

From the SEM micrographs of the worn surfaces (Figure 8), the addition of Ti can be seen to trigger the delamination mechanism, even in low normal loads. The degree of crack formation in 0Ti and 0.15Ti alloys is low, while the 0.75Ti and 1.5Ti (Figure 8E–H) showed cracks that were formed in transverse and parallel directions relative to the wear tracks. Propagation of longitudinal and transverse cracks resulted in the delamination of layers from the worn surface based on the formation of flaky debris, as demonstrated in Figure 9D.

The SEM micrographs of wear debris, as presented in Figure 9, indicate a ribbon shape and corrugated structure, along with crushed debris for 0Ti and 0.15Ti, which imply an abrasive mechanism (Figure 9A,B). Furthermore, the parallel wavy fringes of ribbon shape debris could be ascribed to the formation of shear bands [37]. The plate-like wear debris, as seen in Figure 9C,D, indicate the delamination of the worn surfaces in 0.75Ti and 1.5Ti samples. In Figure 9D, the 1.5Ti sample consisted of a higher fraction of plate-like debris caused by an intensive delamination.

### 3.3. Electrochemical Tests

#### 3.3.1. Electrochemical Impedance Spectroscopy

**Figure 10:**
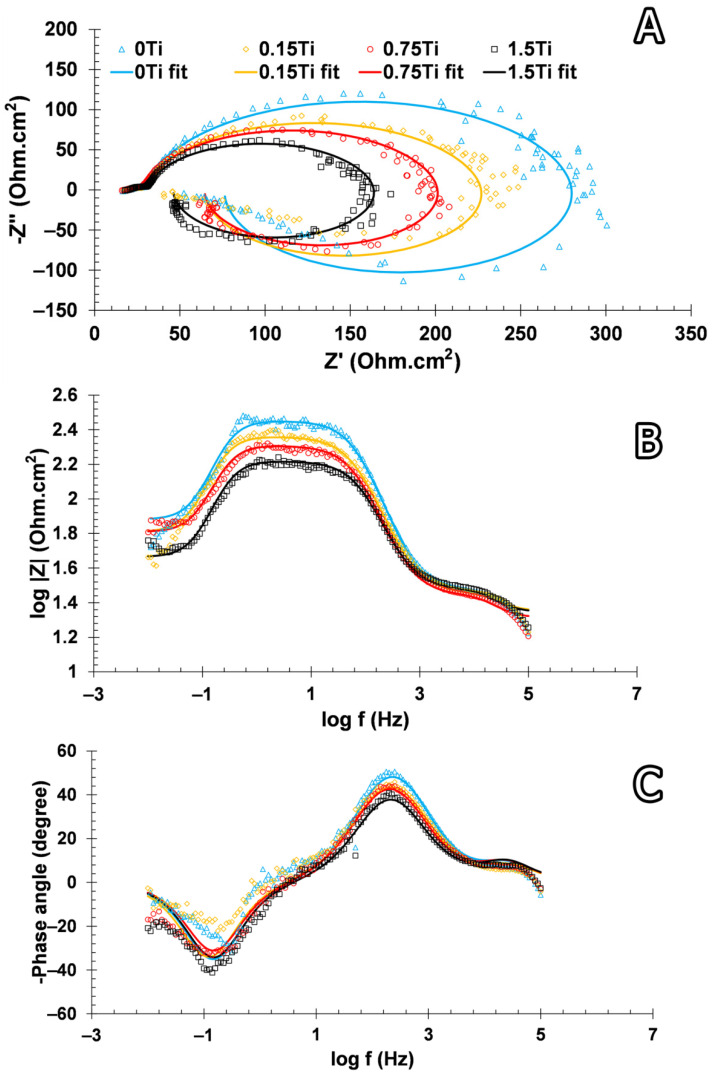
(**A**) Nyquist, (**B**) Bode modulus, and (**C**) Bode-phase angle curves obtained from EIS measurements (markers and solid lines represent the experimental Z observations and the fitted data, respectively).

To probe the corrosion resistance of the prepared alloys, electrochemical EIS and polarization techniques were employed. In the EIS technique, a limited sinusoidal potential with variable frequencies was applied to the system and the responses were collected as the real (Z′) and imaginary (Z″) impedance. Nyquist and Bode plots are two well-known plotting methods used to represent EIS results (Figure 10).

**Figure 11:**
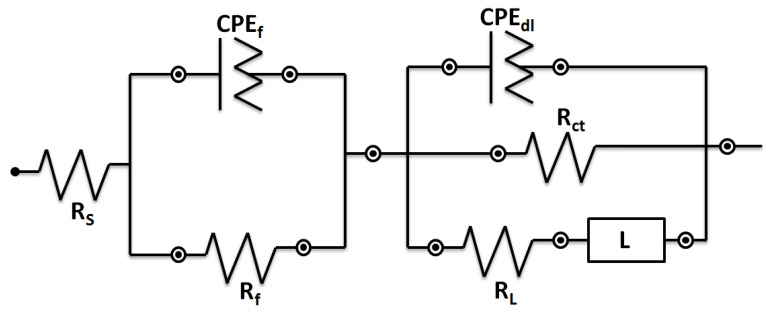
The electrical equivalent circuit utilized to fit the EIS experimental data.

The diameter of the Nyquist plots arguably indicates the corrosion resistance of a system [38]. The outcomes indicated that the Nyquist diameter of the samples diminished in the following order 0Ti > 0.15Ti > 0.75Ti > 1.5Ti. This can be related to the more compact passive layer in 0Ti and 0.15Ti samples compared to the other ones. The higher and lower impedance moduli in almost all frequencies were observed for 0Ti and 1.5Ti samples, respectively (Figure 10B). In addition, the higher-phase angle value in 0Ti at the maximum of the peak, which can be observed in the Bode-phase angle curve, further demonstrates the creation of a greater protective oxide layer on the alloy surface. The inductive loop that appeared at lower frequencies can be introduced to the adsorption–desorption of the ions present in the electrolyte on the electrode surface. The electrical equivalent circuit can usefully interpret electrochemical analyses [39]. Thus, an inductance containing an electrical equivalent circuit was applied to adjust the EIS experimental data (Figure 11).

**Figure 12:**
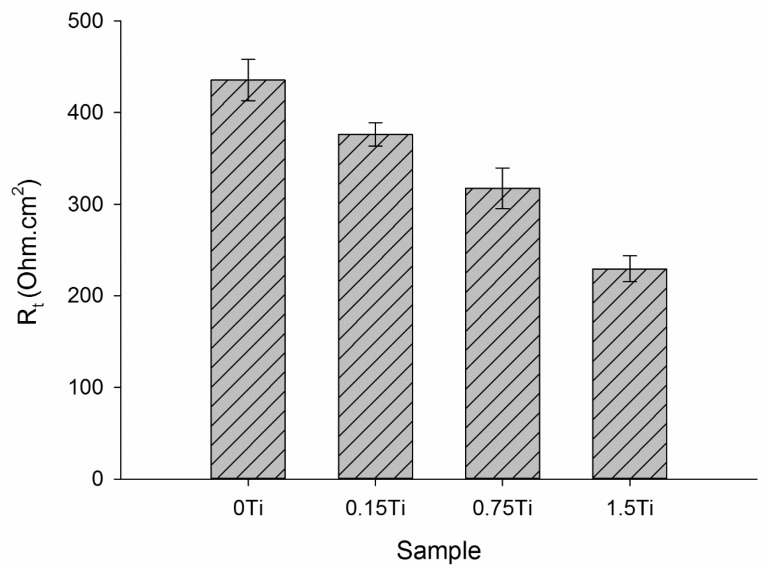
The total resistance values of the samples.

In the electrical equivalent circuit depicted in Figure 11, R_s_, R_f_, R_ct_, and R_L_ are the solution, passive layer, charge transfer, and inductive resistances, respectively. CPE_dl_ and CPE_f_, in relation to the double layer and the passive film, are constant phase elements. L is the inductance element to model the inductive loop in the Nyquist plots. In this equivalent circuit, CPE was applied instead of the pure capacitance to better model the experimental data. The impedance value of the constant phase element is obtained from Z = 1/(Y0jω)^n^, where C is the capacitance, ω is the phase, Y0 is the admittance, and j is the imaginary expression. The value of n (0–1) is related to the electrode surface roughness. When the value of n is equal to 0 and 1, the impedance of the CPE can be related to an ideal resistance and an ideal capacitor, respectively. The experimental data were modelled using ZsimpWin software. Figure 10 shows that the modeling results agree with the experimental measurements, and Table 2 shows the values of elements in the equivalent circuit. The total resistance (R_t_ = R_f_ + R_ct_ + R_L_) value was considered as the electrode corrosion resistance, as represented in Figure 12.

According to Figure 12, the highest and the lowest total resistance belonged to 0Ti and 1.5Ti samples, respectively; this was also previously observed in the Nyquist and Bode plots. These results could be related to the protection of the passive layer on the electrode surface. In fact, increasing the concentration of Ti element in the alloy led to the creation of galvanic cells in the working electrode and negatively affected the alloy resistance against corrosion.

#### 3.3.2. Potentiodynamic Polarization Test

**Figure 13:**
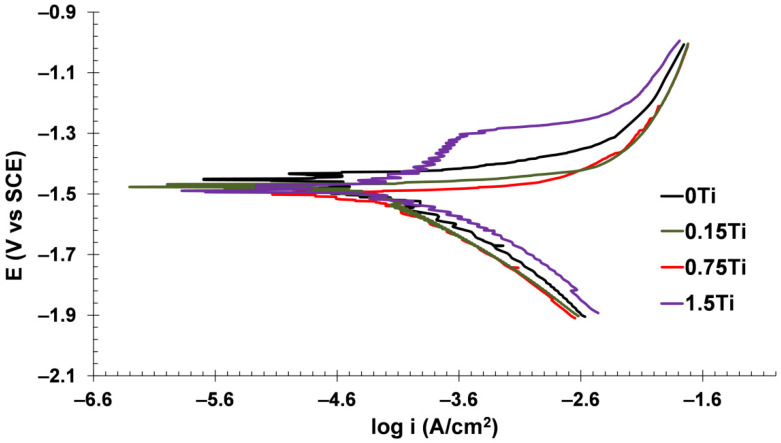
Polarization curves of the studied samples.

The polarization technique is another efficient method to study the electrochemical reactions of a system. The polarization plots of samples are given in Figure 13 and the Tafel extrapolated outcomes are summarized in Table 3.

In Table 3, β_a_ and β_c_ are anodic and cathodic Tafel slopes, while E_corr_ and i_corr_ are the corrosion potential and corrosion current density, respectively. Polarization resistance was obtained from the Stern–Geary equation [40]:

(1) $$R_{p}=(\beta_{a}\;\cdot \;\beta_{c})/(2.303\;\cdot {\;i}_{\mathrm{corr}}\;\cdot \;(\beta_{a}+\beta_{c}))$$

According to Table 3, raising the Ti content diminished the polarization resistance and increased the corrosion current density. As demonstrated in the EIS results, the galvanic corrosion between the Ti particles dispersed in the Mg-5Sn matrix decreased the protection performance of the oxide layer coated on the electrode surface. The increment of absolute values of the corrosion potential (E_corr_) in the samples with higher Ti concentrations confirms the thermodynamic tendency of the electrode to corrode reactions in the corrosive environment. As illustrated in the anodic branch of the polarization plot in the 1.5Ti sample, there is critical potential at about −1.3 V where the current density increased rapidly. In fact, in this critical potential, the passive layer on the working electrode was broken owing to the initiation of pitting corrosion in the presence of Cl^−^ anions adsorbed on the oxide layer. The galvanic cells were formed after doping Ti particles in the Mg-5Sn matrix created pitting susceptible sites on the surface of the used electrode. Consequently, it can be concluded from the polarization analysis that the presence of Ti in the Mg-5Sn matrix does not promote the corrosion resistance of the electrode, but also may create susceptible sites for pitting corrosion.

#### 3.3.3. Corrosion Microstructure

**Figure 14:**
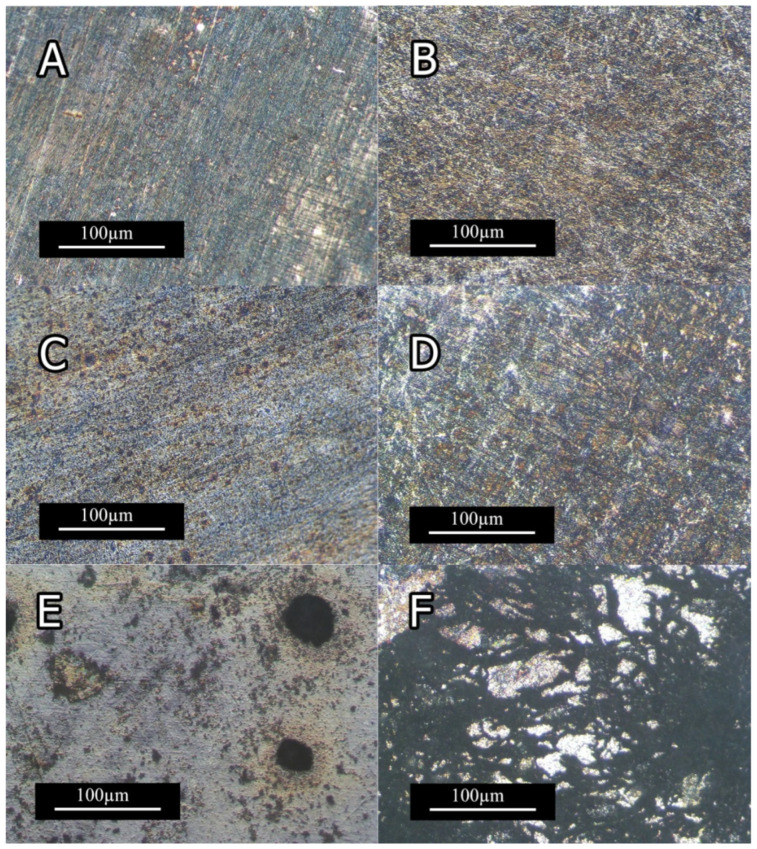
Optical micrographs of the samples’ surface after immersion in 3.5 wt.% NaCl solution (**A**) 0Ti after 10 min; (**B**) 0Ti after 2 h; (**C**) 0.15Ti after 10 min; (**D**) 0.15Ti after 2 h; (**E**) 1.5Ti after 10 min; and (**F**) 1.5Ti after 2 h.

**Figure 15:**
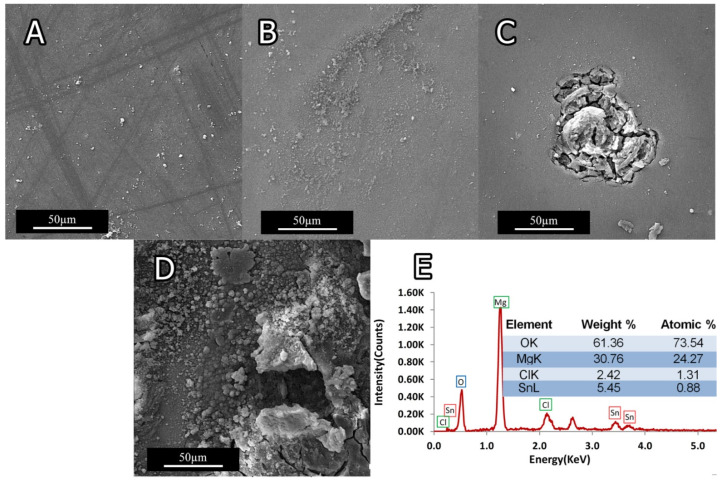
SEM micrographs of sample’s surface after immersion in 3.5 wt.% NaCl solution: (**A**) 0Ti after 10 min immersion; (**B**) 0Ti after 2 h immersion; (**C**) 1.5Ti after 10 min immersion; and (**D**) 1.5Ti after 2 h immersion. (**E**) The corresponding EDS analysis.

Figure 14 and Figure 15 depict the optical micrographs and SEM micrographs of corrosion morphologies of extruded Mg-5Sn-XTi alloys, respectively. As shown in Figure 14 A,C, 0Ti and 0.15Ti samples were not considerably affected by solution up to 10 min, while the 1.5Ti sample showed corrosion spots, indicating the nucleation of pitting corrosion at the same time step. With a further increase in immersion time, corrosion spots were formed on 0Ti and 0.15Ti samples, whereas 0.75Ti and 1.5Ti samples exhibited severe corrosion features with cluster pitting spots and filiform corrosion (Figure 14B,D,F). The presence of Ti facilitated the initiation and evolution of pitting corrosion given in Figure 14E. The uneven distribution of corrosion spots can be a result of the inhomogeneous distribution of Mg_2_Sn, SnTi_3_, and/or Ti particles in the Mg-5Sn matrix.

Figure 15A,B show the SEM images of 0Ti sample’s surface, indicating negligible effect of NaCl solution up to 10 min, while the surface was uniformly covered by the gray corrosion product after 2 h. Pitting state can be observed in Figure 15C,D; in these samples, the corrosion attack went deep into the matrix due to the presence of Ti.

A typical EDS analysis of the corroded surfaces (Figure 15E) discloses that the main constituents are Mg, Cl, and Sn. With respect of Mg to O_2_ ratios, it can be inferred that Mg(OH)_2_ was the main constituent of corrosion product. The corrosion reaction can occur following oxidation of *Mg* as *Mg*^+2^ and *H*_2_*O* to *OH*^−^. The following reaction describes the Mg-Sn system corrosion:

(2) $$Mg+2H_{2}O\to Mg{\left(OH\right)}_{2}+H_{2}↑$$

The galvanic effects of Mg_2_Sn precipitate accelerated alloy corrosion, which led to the formation of *SnH*_4_, as described below:

(3) $$2Mg+Sn+H_{2}O\to SnH_{4}+Mg{\left(OH\right)}_{2}$$

As the reaction went on, Sn concentrates on the surface of the samples increased, leading to the formation of *SnO*_2_, as described in Equation (4) [41]:

(4) $$SnH_{4}+2H_{2}O\to +Sn{\left(O\right)}_{2}+3H_{2}↑$$

**Figure 16:**
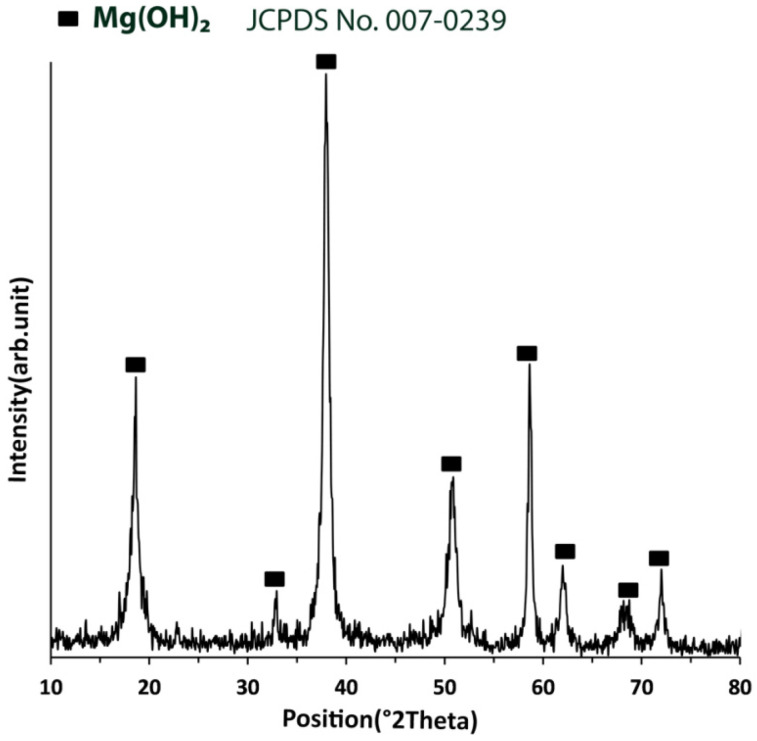
The XRD pattern of the corrosion product of the 0.75Ti sample following immersion in 3.5 wt.% NaCl solution.

The XRD pattern for the corrosion product of the 0.75Ti sample can be developed following immersion in 3.5 wt.% NaCl solution, as shown in Figure 16. All the samples showed similar corrosion product XRD patterns, which mainly consisted of Mg(OH)_2_. According to the mechanism described by Equations (2) and (3), the corrosion reaction started with oxidation of Mg to Mg^2+^, and, subsequently, Mg(OH)_2_ was formed on the surface of the samples. Due to the insolubility of Mg(OH)_2_ in solution, it accumulated on the surface, forming a corrosion layer.

As illustrated in Figure 15, the preferential attack along the Mg-5Sn matrix can be ascribed to the presence of Ti, SnTi_3_, and Mg_2_Sn, which induced the galvanic effect. Investigating the impact of reinforcement particles and second phases on the corrosion properties of Mg alloys reveals that second phases and particles can deteriorate the corrosion resistance of Mg alloys. It has also been reported that increasing the volume fraction of the second phase can decrease the corrosion resistance [42,43,44]. The presence of Ti particles, together with SnTi_3_ and Mg_2_Sn phases, which have different electrochemical potential values, can act as a cathode and lead to the formation of microgalvanic cells, which are responsible for pitting corrosion in Mg-5Sn samples containing Ti as a microalloying element.

## 4. Conclusions

Adding Ti to the Mg-5Sn matrix considerably affected the wear performance of the alloy. Weight loss decreased as the Ti content increased, while the coefficient of friction was enhanced by adding the Ti content.

The sample with 0.75 wt.% Ti showed slight higher wear resistance under 6 and 10 N normal forces, while the sample with 0.15 wt.% Ti showed improved wear resistance under the normal force of 20 N.

SEM images of the worn surfaces indicated oxidation and abrasion wear mechanism in the samples with 0 and 0.15 wt.% Ti, while delamination was found to be the dominant wear mechanism in the sample with 0.75 and 1.5 wt.%.

EIS results showed that the impedance at the lowest frequency as well as the Nyquist diameter values decreased by increasing the concentration of Ti element in the Mg-5Sn-xTi alloys. It was concluded that the addition of Ti can enhance the possibility of pitting corrosion due to the fact that Ti can act as cathode to accelerate the corrosion of the matrix. In light of electrochemical analyses, the extruded Mg-5Sn alloy showed optimal corrosion properties with the highest charge transfer resistance (253.2 Ohm.cm^2^) and the lowest corrosion current density (43.92 μA/cm^2^) among all series.

## Figures and Tables

**Table 1:** Chemical composition (wt.%) of the alloys.

Sample ID	Nominal Composition wt.%	Measured Composition wt.%							
		Sn	Ti	P	Al	Fe	Na	Mn	Mg
0Ti	Mg-5Sn	5.00 ± 0.04	0	0.01	0.15 ± 0.02	0.02	0.17 ± 0.01	0.01	Bal.
0.15Ti	Mg-5Sn-0.15Ti	4.91 ± 0.92	0.11 ± 0.02	0.01	0.14 ± 0.01	0.02	0.16 ± 0.01	0.01	Bal.
0.75Ti	Mg-5Sn-0.75Ti	4.98 ± 0.06	0.81 ± 0.01	0.01	0.13 ± 0.02	0.02	0.17 ± 0.02	0.02	Bal.
1.5Ti	Mg-5Sn-1.5Ti	5.04 ± 0.07	1.46 ± 0.04	0.01	0.15 ± 0.02	0.02	0.17 ± 0.02	0.01	Bal.

**Table 2:** The values of elements in the equivalent circuit shown in Figure 11.

Sample ID	R_s_ (Ohm.cm^2^)	R_f_ (Ohm.cm^2^)	CPE_f_ (S.secn/cm^2^)	n1	CPE_dl_ (S.secn/cm^2^)	n2	R_ct_ (Ohm.cm^2^)	R_L_ (Ohm.cm^2^)	L (Henri.cm^2^)
0Ti	22.31	6.72	1.06 × 10^−6^	1	1.66 × 10^−5^	0.90	253.2	175.4	57.8
0.15Ti	22.54	5.75	1.08 × 10^−6^	1	2.45 × 10^−5^	0.88	201	169.4	44.3
0.75Ti	20.62	6.86	1.07 × 10^−6^	1	2.60 × 10^−5^	0.89	175.8	134.7	46.84
1.5Ti	22.22	7.96	9.27 × 10^−7^	1	2.70 × 10^−5^	0.90	135.1	86.4	18.4

**Table 3:** Electrochemical parameters driven from Tafel extrapolation of the polarization tests.

Samples	β_a_(v·dec^−1^)	−β_c_(v·dec^−1^)	E_corr_ SCE (V)	i_corr_ (μA/cm^2^)	R_p_ (Ohm.cm^2^)
0Ti	0.062	0.244	−1.45	43.92	488.74
0.15Ti	0.054	0.305	−1.47	56.46	352.81
0.75Ti	0.068	0.322	−1.50	75.74	321.85
1.5Ti	0.089	0.326	−1.50	113.02	268.59

## Data Availability

The data presented in this study are available on request from the corresponding author. The data are not publicly available due to its large size and specific file format.
